# 应用于原代T细胞酪氨酸磷酸化蛋白质组的高灵敏度分析方法

**DOI:** 10.3724/SP.J.1123.2024.01016

**Published:** 2024-07-08

**Authors:** Fuchao LIANG, Mi KE, Ruijun TIAN

**Affiliations:** 南方科技大学理学院化学系,广东 深圳 518055; Department of Chemistry, College of Science, Southern University of Science and Technology, Shenzhen 518055, China

**Keywords:** 纳升液相色谱-串联质谱, 酪氨酸磷酸化, 原代T细胞, 共刺激, 信号转导, nanoscale liquid chromatography-tandem mass spectrometry (nanoLC-MS/MS), tyrosine phosphorylation, primary T cell, co-stimulation, signal transduction

## Abstract

酪氨酸磷酸化在T细胞的信号转导过程中发挥着重要的作用,然而其丰度较低,鉴定困难。生物组织样本中分离获得的原代T细胞数量较少,培养和扩增的难度较大,因而常使用永生化细胞系来研究T细胞的酪氨酸磷酸化介导的信号转导过程,这通常会导致获得与原代T细胞相差较大的结论。因此,本研究发展了一种解析原代T细胞酪氨酸磷酸化修饰信号的高灵敏度的蛋白质组学方法。首先,针对原代T细胞数量有限的问题,本研究优化了一套从小鼠脾脏中分离、活化和扩增T细胞的完整流程,第4天时T细胞数量扩增到7倍以上;其次,针对酪氨酸磷酸化修饰丰度较低、不易被质谱检测的难题,本研究利用SH2超亲体(SH2-superbinder)亲和富集和固定化钛离子亲和色谱(Ti^4+^-IMAC)技术对抗CD3和抗CD28单克隆抗体共刺激条件下的原代T细胞多肽样品进行了酪氨酸磷酸化多肽富集,并结合纳升液相色谱-串联质谱(nanoLC-MS/MS)进行解析。最终成功从1 mg蛋白质中鉴定到282个酪氨酸磷酸化位点,其中包含T细胞受体膜蛋白CD3胞内区的免疫受体酪氨酸激活序列(ITAM)上的多个酪氨酸磷酸化位点,以及信号转导相关蛋白ZAP70、LAT、VAV1等的重要位点信息。综上,本研究发展了一套深度解析原代T细胞中的酪氨酸磷酸化修饰的高灵敏度蛋白质组学分析流程,有望应用于绘制更接近生理状态下的信号转导网络。

## Abstract (en)

Tyrosine phosphorylation, a common post-translational modification process for proteins, is involved in a variety of biological processes. However, the abundance of tyrosine-phosphorylated proteins is very low, making their identification by mass spectrometry (MS) is difficult; thus, milligrams of the starting material are often required for their enrichment. For example, tyrosine phosphorylation plays an important role in T cell signal transduction. However, the number of primary T cells derived from biological tissue samples is very small, and these cells are difficult to culture and expand; thus, the study of T cell signal transduction is usually carried out on immortalized cell lines, which can be greatly expanded. However, the data from immortalized cell lines cannot fully mimic the signal transduction processes observed in the real physiological state, and they usually lead to conclusions that are quite different from those of primary T cells. Therefore, a highly sensitive proteomic method was developed for studying tyrosine phosphorylation modification signals in primary T cells. To address the issue of the limited T cells numbers, a comprehensive protocol was first optimized for the isolation, activation, and expansion of primary T cells from mouse spleen. CD3^+^ primary T cells were successfully sorted; more than 91% of the T cells collected were well activated on day 2, and the number of T cells expanded to over 7-fold on day 4. Next, to address the low abundance of tyrosine-phosphorylated proteins, we used SH2-superbinder affinity enrichment and immobilized Ti^4+^affinity chromatography (Ti^4+^-IMAC) to enrich the tyrosine-phosphorylated polypeptides of primary T cells that were co-stimulated with anti-CD3 and anti-CD28. These polypeptides were resolved using nanoscale liquid chromatography-tandem mass spectrometry (nanoLC-MS/MS). Finally, 282 tyrosine phosphorylation sites were successfully identified in 1 mg of protein, including many tyrosine phosphorylation sites on the immunoreceptor tyrosine-based activation motif (ITAM) in the intracellular region of the T cell receptor membrane protein CD3, as well as the phosphotyrosine sites of ZAP70, LAT, VAV1, and other proteins related to signal transduction under costimulatory conditions. In summary, to solve the technical problems of the limited number of primary cells, low abundance of tyrosine-phosphorylated proteins, and difficulty of detection by MS, we developed a comprehensive proteomic method for the in-depth analysis of tyrosine phosphorylation modification signals in primary T cells. This protocol may be applied to map signal transduction networks that are closely related to physiological states.

T细胞是免疫系统的重要组成部分,在杀伤肿瘤细胞及维持机体稳态等方面发挥着重要作用^[1]^。酪氨酸磷酸化深度参与T细胞的免疫应答过程,全面解析T细胞的酪氨酸磷酸化蛋白质组学,对于深入了解免疫调节机制、优化肿瘤免疫疗法等诸多方面具有重要意义^[2]^。

基于质谱的蛋白质组学技术的发展使得酪氨酸磷酸化位点的大规模发现成为可能^[3⇓⇓-6]^。然而,酪氨酸磷酸化具有较低的化学计量学特征,仅占所有磷酸化的0.1%,其位点的富集往往需要mg级别的蛋白质起始量^[7]^。目前针对酪氨酸磷酸化位点的富集,主要包括抗体亲和富集与金属离子亲和色谱(immobilized metal ion affinity chromatography, IMAC)或金属氧化物亲和色谱(metal oxide affinity chromatography, MOAC)联用的策略^[8]^。Kim等^[9]^在抗CD3和抗CD28单克隆抗体单独刺激或共刺激条件下,利用抗体亲和富集与IMAC串联的方法,鉴定并量化了Jurkat T细胞在不同刺激条件下的101个酪氨酸磷酸化位点,构建了不同抗体刺激条件下T细胞酪氨酸磷酸化信号转导网络。但是,基于抗体的酪氨酸磷酸化富集策略,往往存在亲和力较小、富集效率低以及实验费用昂贵等缺点。SH2超亲体(SH2-superbinder)是一种可以与磷酸化酪氨酸高度亲和的蛋白结构域,由Kaneko等^[10]^对蛋白酪氨酸激酶Fyn的SH2结构域突变3个位点而获得。与正常生理状态下的SH2结构域相比,该突变体对于酪氨酸磷酸化位点的亲和力提高了380倍。Bian等^[11]^利用该结构首先开发了SH2超亲体和Ti^4+^-IMAC联用的高效富集方法,从9种永生化的细胞系(各5 mg蛋白质)中解析了10030个酪氨酸磷酸化位点,为大规模解析酪氨酸磷酸化位点奠定了方法学基础;Dong等^[12]^进一步借助该方法,以5 mg和2 mg的蛋白质起始量从未经处理的和经酪氨酸磷酸酶抑制剂(原钒酸钠)处理的Jurkat T细胞裂解液中分别鉴定到343个和1800多个酪氨酸磷酸化位点,该方法与基于抗体的富集方法相比,鉴定深度得到了大大提升。

虽然上述研究利用永生化的细胞系,通过高效富集策略成功解析了许多T细胞信号转导过程中的关键信号分子,对T细胞认识及应用带来了新的观点。然而,永生化细胞系的蛋白质丰度和翻译后修饰与活体动物的原代细胞差异较大,不具有不同组织脏器所携带的时空特异性信息^[13]^。此外,生物组织样本分离获得的原代T细胞数量十分有限。这些问题极大地增加了原代T细胞酪氨酸磷酸化信号事件的解析难度。 因此,发展一种研究原代T细胞的酪氨酸磷酸化的方法,对于理解更接近真实生理状态的免疫调节机制、优化基于T细胞的免疫治疗策略等十分重要。

针对上述问题,本研究首先建立了一套完整的原代T细胞培养流程,实现了小鼠脾脏原代T细胞的分离、活化和体外扩增培养;并利用SH2超亲体和Ti^4+^-IMAC对酪氨酸磷酸化位点高选择性和高灵敏度富集的特点,对抗CD3和抗CD28单克隆抗体共刺激5 min条件下的原代T细胞的酪氨酸磷酸化多肽进行了高效富集;最后,结合纳升液相色谱-串联质谱(nanoLC-MS/MS)技术,深度解析并定量了原代T细胞的酪氨酸磷酸化位点信息(图1)。为后续解析原代T细胞在各种复杂条件下的信号转导网络奠定了方法基础,并提供了数据支持。

**图1:**
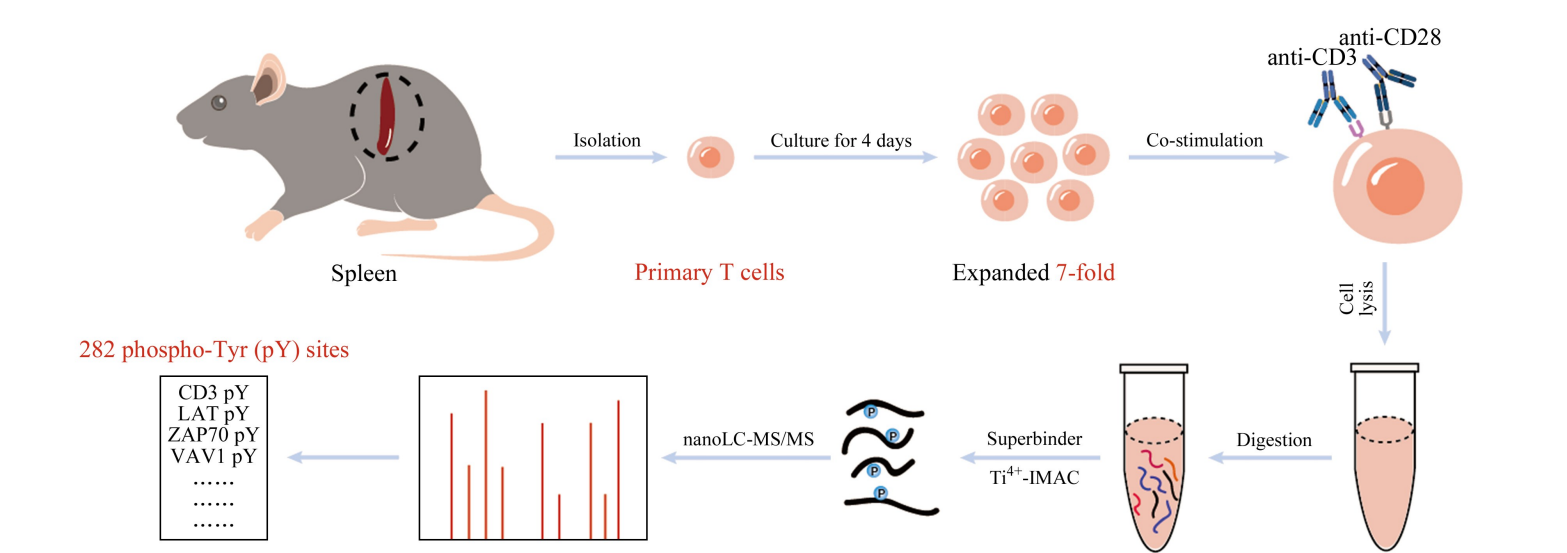
原代T细胞共刺激条件下的酪氨酸磷酸化蛋白质组分析流程

## 1 实验部分

### 1.1 仪器、试剂与材料

Easy nano-LC1200高效液相色谱仪和Q-Exactive HF-X高分辨质谱仪购自Thermo Fisher公司,JY 96-IIN超声波细胞粉碎仪购自宁波新芝公司,高速冷冻离心机购自Eppendorf公司。

三氟乙酸(TFA, HPLC级)、二硫苏糖醇(DTT)、碘代乙酰胺(IAA)和胰蛋白酶(trypsin, T1426)均购自Sigma公司。链霉素-青霉素混合液(30-002-CI)、RPMI 1640培养基(10-040-CV)和磷酸盐缓冲液(21-040-CV)购自康宁公司。胎牛血清(FBS)购自GIBCO公司,抗CD3单克隆抗体(145-2C11)和抗CD28单克隆抗体(BE0015-1-1MG)购自eBioscience公司,小鼠CD3^+^T细胞分选试剂盒(19851RF)购自STEMCELL公司,抗ERK1/2单克隆抗体(4695)、抗磷酸化的ERK1/2抗体(9101)购自Cell Signaling Technology公司,辣根过氧化物酶标记山羊抗兔IgG(H+L)的二抗(A0208)购自碧云天公司,小鼠重组白细胞介素-2(IL-2, 212-12-5)购自Peprotech公司,CD3-BV510流式抗体(100353)、CD25-PE流式抗体(553075)和流式染色缓冲液(554657)购自BD公司,Pierce^TM^ 660 nm蛋白定量试剂盒(22660)购自Thermo Fisher公司。聚偏二氟乙烯膜(PVDF, IPFL00010)购自Millipore公司,C18 SPE除盐柱(SBAN-WAT054960)购自Waters公司。

SPF级C57BL/6J小鼠(6~8周),体重20~25 g,购自广东药康生物科技有限公司,于南方科技大学实验动物中心饲养,饲养环境适宜,12 h/12 h的明暗交替,保持自由进食和饮水。本研究经南方科技大学实验动物伦理委员会批准(编号:SUSTech-JY202201025),实验流程符合《实验动物管理条例》。

### 1.2 脾脏CD3^+^T细胞的分离培养

#### 1.2.1 脾脏CD3^+^T细胞的磁珠分选

对C57BL/6J小鼠进行脱颈椎处死,迅速置于75%的酒精中进行表面消毒。使用灭菌器械迅速将小鼠的脾脏解剖取出,将脾脏组织置于4 ℃预冷的磷酸盐缓冲液中备用。

将70 μm的细胞筛网置于50 mL离心管上,用1 mL冰冷的磷酸盐缓冲液对筛网进行润洗;将解剖出的脾脏组织置于细胞筛网上,使用注射器活塞研磨,同时向筛网上方加入预冷的磷酸盐缓冲液润洗,直至组织完全磨碎。将组织研磨液以400 g离心5 min,吸弃上清液。使用磷酸盐缓冲液将细胞进行重悬,使细胞密度达到1×10^8^ cell/mL。按照STEMCELL公司的小鼠CD3^+^T细胞分选试剂盒说明书对CD3^+^T细胞进行分选,吸取分离得到的细胞,以400 g离心5 min,弃去上清液。

#### 1.2.2 原代T细胞的活化培养

配制T细胞培养基,其主要成分为RPMI 1640空白培养基、10%(v/v)胎牛血清、1%(v/v)链霉素-青霉素混合液、50 μmol/L *β*-巯基乙醇和2 ng/mL IL-2。

对培养T细胞的孔板进行抗体包被,用磷酸盐缓冲液对抗CD3和抗CD28抗体进行稀释,使两种抗体的最终质量浓度均为1 μg/mL,将配制好的抗体稀释液置于24孔板中,每孔1 mL, 4 ℃孵育过夜(14 h以上)。

弃去24孔板中的抗体稀释液,然后在每孔中种入1 mL密度为1×10^6^ cell/mL的细胞。活化2天后,将细胞收集到离心管中,以500 g离心10 min,将细胞用配制好的T细胞培养基重悬,进行扩增培养。

### 1.3 流式细胞术检测

使用细胞计数板对扩增得到的细胞进行计数,取1×10^6^个细胞,以500 g离心5 min,收集细胞沉淀,加入1 mL流式染色缓冲液吹打混匀,以400 g离心5 min,弃去上清液,加入80 μL流式染色缓冲液进行重悬,加入2 μL CD3-BV510或CD25-PE抗体混匀,室温避光孵育20 min。随后加入2 mL流式染色缓冲液轻柔洗涤细胞,以500 g离心5 min,弃去上清液,使用500 μL流式染色缓冲液重悬,上机检测。

### 1.4 原代CD3^+^T细胞的抗体刺激

将培养4天后的T细胞离心收集,使用不含FBS的RPMI 1640空白培养基对细胞饥饿处理4~6 h,重悬并计数,使每个样本细胞数量为1×10^8^个(对照组3组,抗体刺激组4组),离心后使用1 mL RPMI 1640空白培养基重悬。放置冰上10 min,向其中加入5 μg/mL的抗CD3和抗CD28单克隆抗体,在37 ℃水浴锅中孵育5 min。孵育结束后,迅速向其中加入5×RIPA裂解缓冲液,置于冰上10 min。使用细胞超声破碎仪对细胞进行超声裂解,超声5 s,停止5 s,有效超声时间30 s以上。超声结束后,在4 ℃条件下以12200 r/min离心10 min,取上清液,采用Pierce^TM^ 660 nm蛋白定量试剂盒对蛋白质进行定量分析。

### 1.5 蛋白免疫印迹检测

使用10%的十二烷基硫酸钠-聚丙烯酰胺凝胶,于每个泳道中加入20 μg蛋白质样品,120 V恒压条件下进行蛋白质分离(90 min), 200 mA恒流2 h将蛋白质转印至0.45 μm孔径的PVDF膜上。PVDF膜用奶粉封闭2 h。使用含有0.1% Tween 20、150 mmol/L NaCl、20 mmol/L Tris-HCl的缓冲盐溶液(TBST)洗涤3次后加入抗磷酸化的ERK1/2抗体,于4 ℃摇床孵育过夜。次日,用TBST缓冲液洗涤3次,每次5 min。然后加入辣根过氧化物酶标记山羊抗兔IgG(H+L)的二抗,室温孵育2 h。二抗孵育结束后,使用TBST洗涤3次,每次5 min。最后向PVDF膜加入增强型化学发光显影液进行显影。

### 1.6 样品处理

#### 1.6.1 蛋白质酶解除盐

从抗CD3和抗CD28两种单克隆抗体刺激后的原代T细胞蛋白裂解液中取出1 mg的蛋白质,使用甲醇-氯仿法进行蛋白质沉淀。使用500 μL含有8 mol/L尿素的50 mmol/L Tris-HCl缓冲液(pH 8.0)对蛋白质沉淀进行复溶。然后加入终浓度为10 mmol/L的DTT(55 ℃, 30 min)和30 mmol/L的IAA(室温,避光,15 min)进行还原烷基化。之后,加入终浓度为30 mmol/L的DTT(室温,30 min)终止反应。进一步,用7倍溶液总体积的50 mmol/L Tris-HCl缓冲液(pH 8.5)稀释样品,并加入终浓度为1 mmol/L的CaCl_2_。最后加入胰蛋白酶(酶与蛋白质的质量比为1∶20),于37 ℃酶解16 h后,向酶解溶液中逐步、少量加入10%(v/v)TFA对多肽进行酸化(pH 2~3)。然后高速离心去除沉淀后,收集多肽上清液后使用SPE除盐柱,按照Waters公司除盐柱说明书的步骤进行多肽除盐。

#### 1.6.2 酪氨酸磷酸化多肽富集

使用免疫亲和纯化缓冲液(50 mmol/L Tris-HCl (pH 7.8)、10 mmol/L Na_2_HPO_4_、50 mmol/L NaCl)复溶多肽,并利用1 mol/L Tris-HCl缓冲液调节pH至7.5左右,向溶解的肽段中加入带有工程化的SH2结构域蛋白的微球孵育过夜。使用冰上预冷的免疫亲和纯化(IAP)缓冲液清洗3次,向其中加入含有500 mmol/L咪唑的磷酸盐缓冲液洗脱2次,最后加入10%甲酸水溶液将多肽酸化至pH 2~3。使用含有10层C18膜片的200 μL移液器枪头进行除盐,除盐产物使用装有Ti^4+^-IMAC填料的200 μL移液器枪头进一步纯化,使用10%氨水进行洗脱。使用装有3层C18膜片的200 μL移液器枪头对洗脱下的酪氨酸磷酸化多肽进行除盐,肽段冻干后,使用12 μL含有4%(v/v)甲酸和5%(v/v)乙腈的水溶液进行复溶。

### 1.7 nanoLC-MS/MS分析

采用Easy nano-LC1200色谱系统,使用内径为100 μm的20 cm自制毛细管分析柱,其中填充0.5 cm C4树脂填料(颗粒尺寸为3 μm,颗粒孔径为12 nm)和19.5 cm C18树脂填料(颗粒尺寸为1.9 μm,颗孔径为12 nm),流速为250 nL/min,流动相A相为0.1%(v/v)甲酸水溶液,B相为含0.1% (v/v)甲酸的90%(v/v)乙腈水溶液。梯度洗脱程序如下:0~2 min, 4%B~8%B; 2~57 min, 8%B~28%B; 57~62 min, 28%B~40%B; 62~64 min, 40%B~97%B; 64~80 min, 97%B。

采用纳流电喷雾离子源,电压1800 V,离子传输毛细管温度为300 ℃,以数据依赖采集模式运行。一级质谱参数:扫描范围为*m/z* 350~1550,分辨率为120000,自动增益控制目标(AGC target)为3×10^6^,最大注入时间为20 ms。二级质谱参数:分辨率为15000, AGC target为1×10^5^,最大注入时间为40 ms。

### 1.8 数据分析

使用MaxQuant (版本1.5.5.1)软件对质谱数据进行分析,使用Uniprot数据库中的Mus musculus数据库(2020年10月6日,86477个蛋白质),胰蛋白酶酶解,半胱氨酸烷基化(C,+57 Da)为固定修饰,酪氨酸磷酸化(Y,+79.97 Da)、甲硫氨酸氧化(M,+16 Da)、肽段N端的乙酰化(N-terminal,+42 Da)、天冬酰胺和谷氨酰胺脱酰胺修饰(N/Q,+0.98 Da)为可变修饰。允许最大漏切位点为2。蛋白质和肽段鉴定的错误发现率(FDR)控制在1%以内。蛋白质鉴定标准为至少检测到2个特征性肽段。使用Perseus(版本1.5.5.3)软件进行数据加工并生成火山图。

## 2 结果与讨论

### 2.1 脾脏原代T细胞分离与质控

首先,使用小鼠CD3^+^T细胞分选试剂盒,对研磨得到的小鼠脾脏单细胞悬液进行分选,获得脾脏来源的原代T细胞。短时间静置后,使用倒置显微镜观察,如图2a所示,原代T细胞形态圆润,大小均一,细胞呈现悬浮状态。使用流式细胞术对收集到的细胞进行表面标志物CD3的检测,以验证分选得到的T细胞纯度。如图2b所示,经过磁珠分选后得到的细胞中,90%以上的细胞为CD3^+^T细胞,纯度较高,说明成功分选到小鼠脾脏来源的原代CD3^+^T细胞,可用于后续扩增培养。

**图2:**
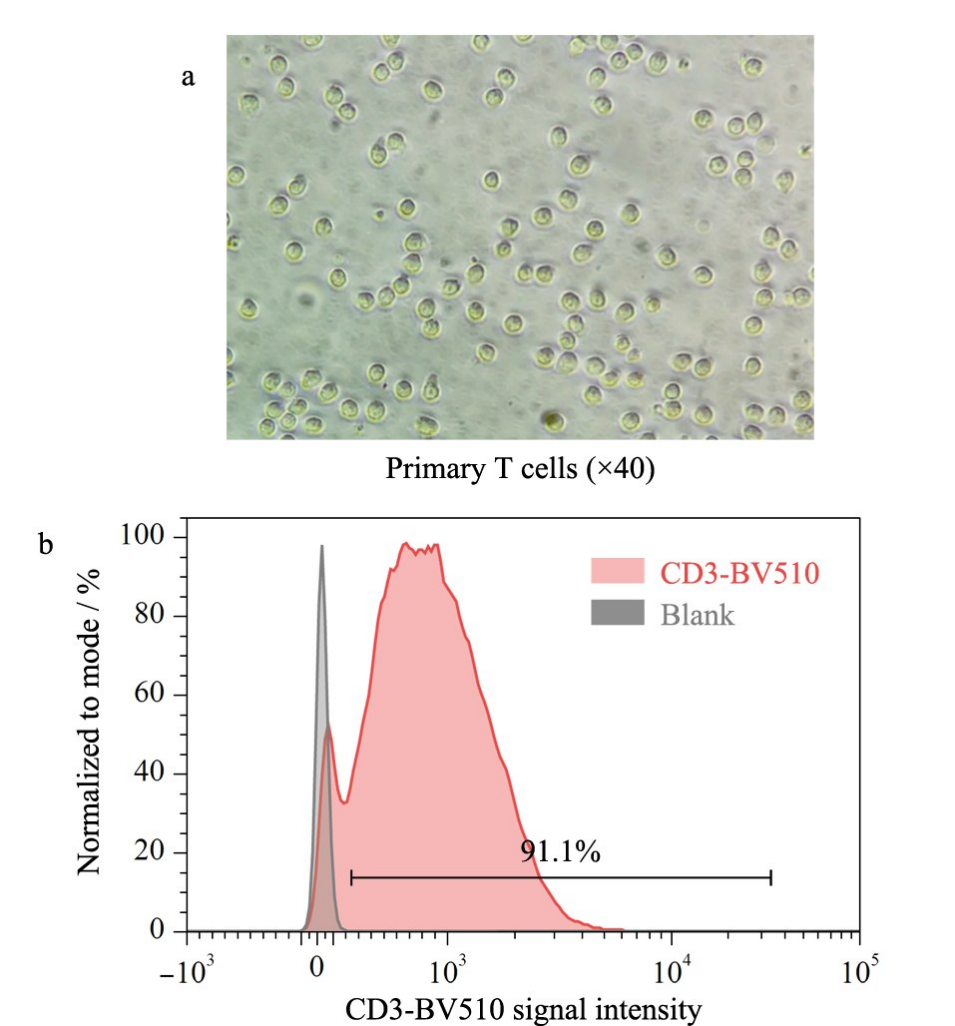
初始分离出的原代T细胞(a)形态和(b)表面标志物检测

### 2.2 T细胞的体外活化及扩增效果评价

为了获得足够数量的细胞用于后续酪氨酸磷酸化富集实验,本研究将对原代T细胞进行活化及扩增培养。T细胞的完全活化需要T细胞受体(TCR)-CD3复合物接受来源于主要组织相容性复合体(MHC)的第一信号、共刺激受体(如CD28)接受的第二信号和多种细胞因子的协同作用^[14]^。分别使用抗CD3和抗CD28单克隆抗体模拟T细胞激活过程中的双重信号,对分离出的原代T细胞进行活化,同时在培养过程中加入细胞因子IL-2促进T细胞增殖。如图3a所示,T细胞在4天的培养过程中不断扩增。第0天时,细胞多呈现单个细胞悬浮状存在,形态均一,圆润饱满;24孔板中活化培养2天后,细胞数量显著增多,细胞之间出现聚团生长现象,团块相对较小;第4天时,细胞大量扩增,数量持续增多,细胞间出现大量聚团现象,团块相对较大,说明其正处于迅速增殖的状态。

**图3:**
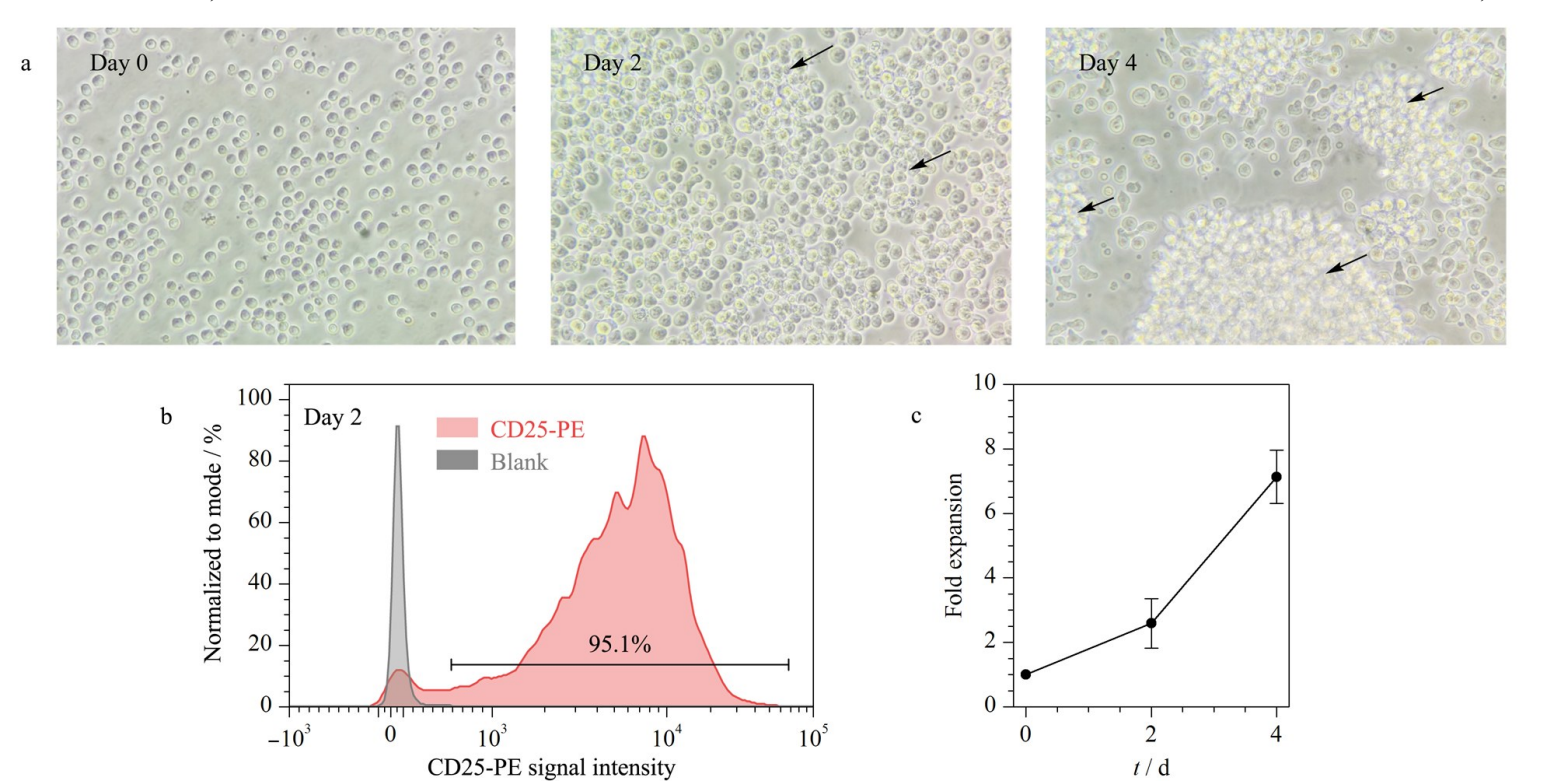
原代T细胞不同培养天数的(a)形态、(b)活化标志物检测和(c)扩增倍数(*n*=5)

CD25是IL-2受体的*α*链,是评估T细胞活化状态的重要标志^[15]^。CD25的维持由IL-2激活信号传导及转录激活蛋白(STAT5)信号所介导,一般在激活后的第2天(48 h)达到峰值^[16]^。为了评估T细胞体外活化效果,通过流式细胞术检测活化后第2天的T细胞CD25的表达情况。如图3b所示,在活化后的第2天,T细胞CD25的阳性率可达95%以上。说明绝大多数细胞IL-2受体被激活,细胞体外活化效果较好。

如图3c细胞扩增曲线显示,第2天时细胞增殖强度相对较小,第4天时细胞总数扩增到起始细胞数量的7倍。且第4天时细胞状态较好。单只小鼠分离培养的脾脏原代T细胞数量(1×10^8^个细胞)即可用于后续的酪氨酸磷酸化蛋白质组学实验。综上,本研究成功建立了小鼠脾脏T细胞的分离、活化以及体外扩增培养的完整流程。

### 2.3 原代T细胞的刺激条件筛选

细胞外调节蛋白激酶(ERK1/2)是丝裂原活化蛋白激酶(MAPKs)的家族成员,其在T细胞接受外来刺激后的激活过中起着至关重要的作用^[17]^,它能够促进转录因子活化蛋白-1(AP-1)的形成,进一步影响IL-2的转录过程。T细胞接受抗CD3和抗CD28单克隆抗体共刺激后,ERK1/2会被显著激活,发生酪氨酸磷酸化^[9]^。为了筛选对小鼠原代T细胞的最佳刺激条件,本部分研究分别使用抗CD3和抗CD28单克隆抗体单独刺激或共刺激3种不同条件进行处理,检测ERK1/2蛋白的磷酸化情况。结果显示,联合使用抗CD3和抗CD28单克隆抗体对T细胞的刺激效果最好,可以充分激活ERK1/2的磷酸化,与文献[9]报道一致。因此,后续实验使用抗CD3和抗CD28单克隆抗体共同刺激原代T细胞,以研究T细胞激活过程中的酪氨酸磷酸化信号转导过程。

### 2.4 双抗体共刺激条件下的原代T细胞酪氨酸磷酸化蛋白质组

在筛选出原代T细胞的最佳刺激条件后,利用SH2超亲体和Ti^4+^-IMAC联用的富集方法,将抗CD3和抗CD28单克隆抗体共刺激5 min条件下的原代T细胞进行了酪氨酸磷酸化富集。基于SH2超亲体和Ti^4+^-IMAC富集策略的独特优势,本研究共鉴定到282个酪氨酸磷酸化位点,其中单个样品的高置信度(probability > 0.75)酪氨酸磷酸化位点鉴定数量都接近250个(图4a)。

**图4:**
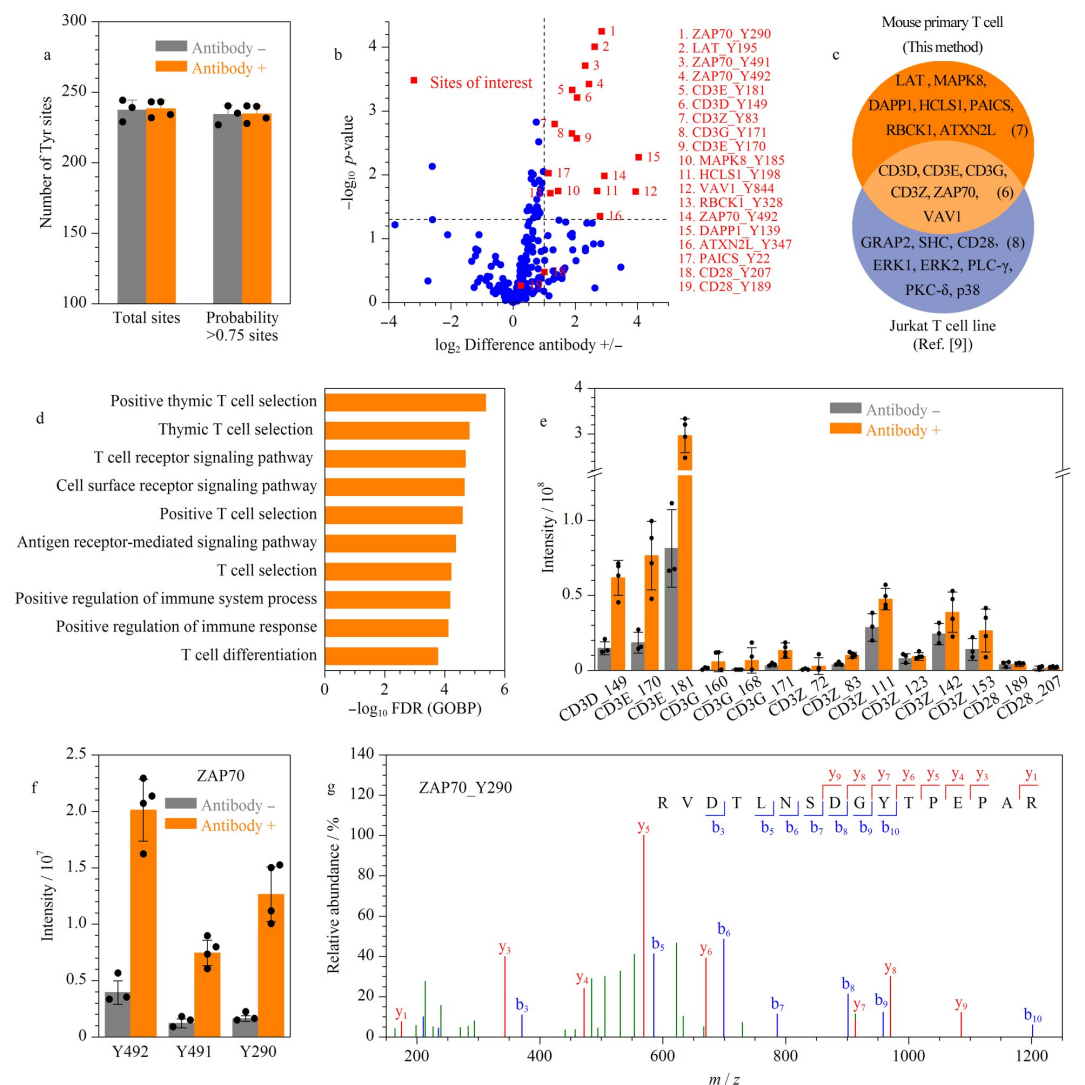
原代T细胞激活条件下酪氨酸磷酸化蛋白质组的分析

T细胞激活过程涉及酪氨酸磷酸化介导的复杂信号转导网络。T细胞接受刺激后,CD3复合物膜内区的免疫受体酪氨酸激活序列(ITAM)酪氨酸残基会发生磷酸化,招募T细胞受体*ζ*链相关蛋白激酶70(ZAP70)等具有SH2结构域的蛋白质发生磷酸化。进一步,T细胞活化连接蛋白(LAT)也被磷酸化,招募其他接头蛋白及效应因子,启动下游信号的激活,并最终引起T细胞增殖^[18]^。火山图展示了原代T细胞激活前后酪氨酸磷酸化位点的差异性变化(图4b),其中包括TCR-CD3信号转导模块*γ*/ε、*δ*/ε和*ζ*/*ζ*上的多个酪氨酸磷酸化位点信息,以及ZAP70、LAT和VAV鸟嘌呤核苷酸交换因子1(VAV1)等T细胞激活过程中关键通路蛋白的酪氨酸磷酸化事件的发生。

虽然科学家们已经借助永生化的Jurkat T细胞,较为详尽地解析了T细胞信号转导过程,但这并不能完全体现真实生理层面下的信号网络。已有研究表明Jurkat T细胞中天然缺乏同源性磷酸酶-张力蛋白(PTEN)和含有SH2结构的5’肌醇磷酸酶(SHIP)两个关键的磷酸酶,并且PTEN的缺失会直接导致磷脂酰肌醇3-激酶(PI3-K)信号通路组成型激活^[19]^。因此,为了比较原代T细胞与Jurkat T细胞在抗CD3和抗CD28单克隆抗体共刺激条件下激活情况的差异,我们将酪氨酸磷酸化上调2倍以上且*p*<0.05的位点对应的蛋白质进行统计,与文献[9]中报道的Jurkat T细胞酪氨酸磷酸化上调2倍以上的位点对应的蛋白质进行了比较,分析了两者之间的异同(图4c)。结果表明,借助小鼠原代T细胞和Jurkat T细胞,两者都能够很好地对CD3信号转导模块、ZAP70和VAV1等与T细胞激活直接相关的重要蛋白质的酪氨酸磷酸化位点差异变化进行全面解析。相比之下,原代T细胞对LAT蛋白的酪氨酸磷酸化位点丰度变化的差异具有更明显的体现。此外,本研究还在原代T细胞中高度灵敏地鉴定到造血系细胞特异蛋白(HCLS1)、丝裂原活化蛋白激酶(MAPK8)以及RanBP型、C3HC4型含锌指蛋白(RBCK1)等多个之前未被广泛报道的蛋白质的酪氨酸磷酸化位点的定量差异变化。这些蛋白质也大都与免疫信号通路的调节相关,例如已有报道指出HCLS1蛋白可能在受体信号传导过程中起重要作用^[20]^。进一步,为了证实本研究流程所获得的数据可靠性,针对原代T细胞在抗体刺激后上调2倍及以上且*p*<0.05的酪氨酸磷酸化位点对应的蛋白质进行了基因本体论(gene ontology, GO)分析(图4d),可以看出其涉及的生物过程大多都与T细胞免疫密切相关,从整体的层面说明数据的真实可靠。

对CD3和CD28的丰度信息进行统计,CD3复合物不同侧链的酪氨酸磷酸化位点在刺激前后的差异变化相对显著,但CD28酪氨酸磷酸化位点在刺激前后变化差异相对较小(图4e),这可能是由于原代T细胞体外培养诱导共抑制受体表达,进而抑制了共刺激受体膜内区的酪氨酸磷酸化^[21]^。该结果体现出原代T细胞与Jurkat T细胞信号转导的差异,也进一步说明研究原代T细胞酪氨酸磷酸化信号的重要性。

蛋白酪氨酸激酶ZAP70是TCR激活过程中的关键调控因子,其功能的缺失会导致严重的联合免疫缺陷病,对其酪氨酸磷酸化位点的研究具有重要意义。研究人员已经通过电喷雾电离质谱和生化手段^[22,23]^发现了T细胞激活过程中ZAP70的多个酪氨酸磷酸化位点,其中包括位于激酶结构域的Y492(对应小鼠Y491)和Y493位点(对应小鼠Y492),以及位于SH2结构域和激酶结构域之间的Y292位点(对应小鼠Y290)。Y492对T细胞激活具有抑制作用,将Y492突变为苯丙氨酸(492F)会使得ZAP70内源性激酶活性增加4倍以上^[24]^;与之相反,Y493对T细胞激活发挥正向调节作用,将Y493转化为苯丙氨酸(493F)则会显著降低ZAP70的激酶活性^[24]^。在质谱分析中连续的两个磷酸化的酪氨酸鉴定比较困难,本研究单次质谱分析即可鉴定到Y491和Y492两个位点(图4f),体现出本研究所建立的技术流程在鉴定酪氨酸磷酸化位点方面的优越性能。Y292在TCR介导的信号转导过程中起抑制作用,将292位点转化为苯丙氨酸(292F)会导致活化的T细胞核内因子(NF-AT)的结构性表达^[25]^。在本研究中,对Y290位点的丰度变化差异也实现了有效检测(图4f),并得到丰富且高质量的谱图信息(图4g)。总之,利用本研究优化的技术流程,在单次实验中就能够有效地鉴定到小鼠原代T细胞激活前后Y491、Y492及Y290这3个磷酸化的酪氨酸位点定量强度的差异性变化,体现出本技术流程高度灵敏的技术特点。

综上,原代T细胞与永生化Jurkat T细胞之间信号转导存在较大的差异,利用原代T细胞解析酪氨酸磷酸化信号转导网络具有十分重要的意义。原代T细胞数量相对较少,直接分离获得的原代T细胞的裂解液往往不足以用于进行酪氨酸磷酸化位点的富集和蛋白质组学分析。本研究建立的完整流程可以用于解析原代T细胞在不同刺激条件下的信号转导过程中酪氨酸磷酸化位点的变化情况,为富集和分析原代细胞在药物处理条件下的酪氨酸磷酸化翻译后修饰信号的变化提供了新的研究思路,为进一步有效地筛选药物靶点、提高药物靶标的针对性和有效性提供了技术支持。

## 3 结论

本研究发展了一种研究原代T细胞酪氨酸磷酸化蛋白质组的高灵敏度分析方法。实现了原代T细胞的分离、活化及扩增培养,通过SH2超亲体和Ti^4+^-IMAC联用的高灵敏度富集策略,借助nanoLC-MS/MS技术解析了T细胞激活过程中的酪氨酸磷酸化位点变化信息,最终从1 mg的原代T细胞的蛋白起始量中解析了包括T细胞受体蛋白CD3的多个侧链以及许多胞内信号转导相关蛋白ZAP70、LAT、VAV1等在内的282个酪氨酸磷酸化位点。

大量的研究表明,酪氨酸磷酸化修饰蛋白是许多药物的重要作用靶点^[26]^。本研究优化的高度灵敏的蛋白质组学技术能够从原代T细胞中获得酪氨酸磷酸化位点信息,将为解析真实的药物靶标及其结合强度等方面带来新的认知。
